# The Song of the Dying Swan

**Published:** 1886-05

**Authors:** 


### EDITORIAL NOTES.

### THE SONG OF THE DYING SWAN.

The *New York Medical Record* gives a dying kick at creation and ''the Congress,'' like the cross wife, who, when no longer able to *speak* a cross word, held up her fingers to indicate ''scissors.'' Speaking of the meeting of the American Medical Association,'' at St. Louis, the *Record* says :

''The attendance was fairly good, but naturally was made up almost entirely of men from the west and southwest. That part of our great country is determined to hold an international congress which shall be truly representative.''

We venture, to suggest, it would be, in the event it were made up entirely of ''men from the west and southwest,'' as nearly ''representative,'' as if made up solely of New York and Philadelphia specialists—the self annointed, as proposed by the immaculate seven wise men from the east.

Speaking of the Association's endorsement of the International Congress Committee's work, and the election of Dr. Davis to the presidency, and Dr. Hamilton to be Secretary-General, the *Record* goes on to say :

''There was no opposition made to these changes, or to the adoption of the present policy. *The much dreaded descent of Philadelphia's delegates upon the association did not occnr*, (italics ours.) All was harmony and smiles.'' (*Record*, May, 8th.)

Well, we should remark ! ''Harmony,'' as to the approval of the committee's work; and ''smiles,'' aye, very smiles, when Dr. John B. Roberts, sub-chairman of the packed delegation from Philadelphia *kicked back*, on being *kicked out of the convention*; fired; ''The descent,'' you see Bro. Shrady, tried to occur, but couldn't. Shoemaker, Atkinson and a few other code-respecting, law-abiding and decency-loving members, were several too many for "Philadelphia's delegates," who, it was decided by the judicial council, were irregularly elected, and therefore, not entitled to seats. Dr. Hays Agnew was the leader of the gang, but seeing which way the cat would jump, he stood from under, and let the crash fall on Dr. J. B. Roberts. Poor Roberts was so flattened out that he "could'nt see his way clear," he said, "to accepting the honor conferred upon him," (secretary to the surgical section) and resigned. "Smiles," brother Shrady ? you are right.

The smiles on the faces were fearful to see,
As the delegates sat down on Dr. J. B.,

(Dr. Merryman suggests us to say.)

